# Real-World Effectiveness of Tezepelumab in Severe Asthma: A Comparative Analysis of High and Low T2 Phenotypes

**DOI:** 10.3390/jpm16030167

**Published:** 2026-03-18

**Authors:** Eusebi Chiner, Ignacio Boira, Mónica Antón, María Ángeles Bernabeu, Celia Cuevas, Paula Fernández, Violeta Esteban, Mónica Llombart

**Affiliations:** 1 Pneumology Department, San Juan de Alicante University Hospital, Ctra N-332, s/n, 03550 San Juan de Alicante, Alicante, Spain; echinervives@gmail.com (E.C.); celia.cuevas@goumh.umh.es (C.C.); paulafernandez1441@gmail.com (P.F.); violeta_er@hotmail.com (V.E.); llombartcanto@gmail.com (M.L.); 2 Department of Clinical Medicine, Miguel Hernández University, 03202 Elche, Alicante, Spain; 3 Allergy Department, San Juan de Alicante University Hospital, 03550 San Juan de Alicante, Alicante, Spain; manton.girones@hotmail.com; 4 Pharmacy Department, San Juan de Alicante University Hospital, 03550 San Juan de Alicante, Alicante, Spain; bernabeu_marmar@gva.es

**Keywords:** severe asthma, uncontrolled severe asthma, tezepelumab, biologic therapy

## Abstract

**Background**: Tezepelumab has demonstrated efficacy in severe uncontrolled asthma (SUA), although real-world evidence remains limited. **Methods**: We included patients with SUA who completed at least 6 months of treatment. Lung function, eosinophil counts, IgE, FeNO, comorbidities, and changes in asthma control were assessed using ACT, ACQ, the VAS, and quality of life (AQLQ), as well as severe exacerbations (hospital admissions and emergency visits), oral corticosteroid (OCS) courses, OCS withdrawal/dose reduction, and reductions in maintenance and reliever medication. Response was evaluated using the FEOS and EXACTO scales. Baseline (T0) and 6-month (T6) outcomes were compared in the overall cohort and according to T2-high (eosinophilic/allergic) vs. T2-low phenotype. **Results**: A total of 33 patients were analyzed (58 ± 12 years; 94% women), with a high burden of comorbidities (88%), mainly rhinosinusitis (79%), obesity (41%), and smoking (37%). Of these, 45.5% had received prior biologic therapy. All patients were on high-dose ICS + LABA, frequently with LAMA and other controllers; 30% were on maintenance OCS. In the previous year, 49% had been hospitalized, 97% had attended the emergency department, and 100% required OCS courses. After 10 ± 3 months, the overall group showed significant improvement in VAS, ACT, ACQ, and AQLQ (*p* < 0.001), with a reduction in eosinophils, but no significant change in FEV1%. Severe exacerbations, emergency visits, hospitalizations, and OCS courses decreased markedly (*p* < 0.001). Among 10 patients on maintenance OCS, OCS were discontinued in 7 and reduced in 3; maintenance/reliever medication was also reduced. The T2-high phenotype showed a higher likelihood of “complete response” (52% vs. 17% in non-T2), although “good response” predominated in non-T2; this difference was significant (*p* = 0.04). **Conclusions**: Tezepelumab improved asthma control and reduced healthcare utilization and corticosteroid use in both T2 and non-T2 patients, achieving clinical remission in 40%, with better outcomes in T2.

## 1. Introduction

Asthma is a complex, heterogeneous, and dynamic respiratory disease characterized by chronic airway inflammation with variable symptoms and expiratory airflow limitation that may lead to progressive airway remodeling [1,2,3].

Severe uncontrolled asthma (SUA) is diagnosed when poor control persists despite high-dose inhaled glucocorticoids (ICS) and long-acting β-adrenergic agonists (LABA) during the last year, or when oral glucocorticoids (OCS) have been required for ≥6 months [2]. Between 5 and 10% of patients with asthma have severe asthma, and nearly 50% are poorly controlled [4]. In Spain, the prevalence of SUA is 3.9% [5].

Patients with asthma are classified according to Th2 activity into T2 (allergic/eosinophilic) and non-T2. Identifying the phenotype in SUA, as well as the endotype based on the existing hypersensitivity mechanism and the assessment of eosinophilia and ex vivo challenge tests, is essential for diagnosis, treatment, and prognosis [6,7]. SUA entails a high burden with a high annual cost per patient [8].

Current treatment follows a stepwise approach based on ICS and LABA. In severe cases, biologic therapies are proposed: anti-IL-5 (mepolizumab, reslizumab), anti-IL-5Rα (benralizumab), anti-IL-4/IL-13 (dupilumab), anti-IgE (omalizumab), and tezepelumab, which acts at the epithelial barrier level [1,2,9]. Prolonged use of OCS is not recommended due to adverse effects and increased mortality risk; therefore, reducing their use is a primary objective [1,2,10,11,12].

Thymic stromal lymphopoietin (TSLP) is a cytokine produced by epithelial cells that initiates and sustains airway inflammation by activating several cell types involved in both T2 and non-T2 asthma [12,13]. Tezepelumab is a fully human monoclonal antibody (IgG2λ) that blocks TSLP [12]. Studies such as NAVIGATOR (phase III) [14] and PATHWAY (phase IIb) [15] showed that it significantly reduces exacerbations regardless of phenotype, biomarkers, or allergic profile [14,15,16,17]. It has also shown efficacy in overlapping phenotypes [16] and in patients with comorbidities. The CASCADE study demonstrated a reduction in eosinophils in the bronchial submucosa and in airway hyperresponsiveness [12].

While mechanisms in T2-high asthma are well characterized, those in T2-low asthma are less well defined. The latter is associated with neutrophilic or paucigranulocytic inflammation, greater corticosteroid resistance, and is linked to obesity, smoking, and older age, with low blood eosinophils and/or low Fractional exhaled nitric oxide (FeNO) [18]. The existence of a truly non-T2 phenotype is debated, since bronchial biopsy studies show that 82% of patients with low blood eosinophils had submucosal eosinophilia [19].

Real-world evidence on tezepelumab remains limited. A multicenter German study including 129 patients stands out [20], as well as a European series of 175 patients reporting clinical remission of <20% in T2-low versus 55% in T2-high [21]. Another retrospective and multicenter study included 103 patients treated with tezepelumab and a follow-up period of one year. The annual exacerbation rate decreased by 66.7% [22].

In Spain, available data come from small studies: 3 patients [23], 9 cases over 7 months [24], 13 patients over 4 months [25], and a multicenter study of 57 patients at 6 months [26]. Recently, a single-center Italian study with 30 patients assessed response at 30 days [27].

Our hypothesis is that tezepelumab is effective in SUA and may reduce exacerbations, oral corticosteroid use, emergency visits/hospitalizations, and improve asthma control and lung function in both T2-high and T2-low phenotypes. The objectives of this study are to evaluate the clinical benefit of adding tezepelumab in patients with SUA in terms of asthma control, lung function, hospitalizations, and emergency visits, in both T2 and non-T2 patients.

## 2. Materials and Methods

### 2.1. Study Design

Ambispective, real-world, single-center observational study based on medical records of patients with SUA who started tezepelumab at a tertiary university hospital, within a joint dispensing protocol with the Hospital Pharmacy Service.

### 2.2. Study Period

January 2024 to December 2025.

### 2.3. Population

All outpatient patients aged ≥ 18 years who were prescribed tezepelumab in the asthma unit by a single specialist were included. Baseline severity was defined according to Spanish guidelines on the management of asthma (GEMA) [2]. Patients were divided into the following groups:

• **Group A:** T2-low phenotype.
• **Group B:** T2-high phenotype (eosinophilic/allergic).

T2-low phenotype was defined as the coexistence of IgE < 100, FeNO < 25 ppb, and <300 blood eosinophils.

### 2.4. Inclusion Criteria

All patients met SUA criteria according to GEMA 5.5 and received 210 mg of tezepelumab subcutaneously every 4 weeks, from T0, for at least 6 months (T6).

Patients were followed periodically in our severe asthma unit prior to starting tezepelumab. Patients received tezepelumab rather than other biologic treatments based on an individualized assessment carried out by our hospital’s biologic therapy committee.

Tezepelumab was administered when patients had at least two exacerbations in the year prior to initiation and a history of poorly controlled asthma symptoms. Exacerbations were defined as loss of control requiring rescue with systemic oral corticosteroids for at least three days and/or emergency department visits and/or hospitalizations due to respiratory symptoms. In patients on maintenance OCS, an exacerbation was defined as doubling the maintenance dose for three days.

All patients were on regular treatment with a high-dose ICS + LABA combination or triple therapy (ICS, LABA, and a long-acting muscarinic antagonist [LAMA]) and could include a leukotriene inhibitor. All used an additional controller (short-acting β2-agonist, SABA) as needed. All patients who received tezepelumab in the asthma unit were included in this study.

### 2.5. Exclusion Criteria

Patients with other respiratory diseases that may share clinical manifestations with severe asthma (vasculitis, chronic cough, etc.) were excluded. Patients with SUA with allergic phenotype or neutrophilic profile were not excluded.

### 2.6. Efficacy Assessment

Asthma symptom control, OCS dose, exacerbations, and forced expiratory volume in 1 s (FEV1) were assessed at baseline and at scheduled visits at 6 and 12 months after initiation. Spirometry was performed according to Spanish Society of Pulmonology and Thoracic Surgery (SEPAR) criteria [28], including reversibility testing in all patients before starting tezepelumab.

For symptom control at baseline and during treatment, the self-administered asthma control test (ACT) questionnaire was used [29]. The minimally important difference for ACT is 3 points. The primary endpoint was good asthma symptom control (ACT ≥ 20). The asthma control questionnaire (ACQ) was also used [29].

Additionally, a visual analog scale (VAS) from 0 to 10 was used, with higher scores indicating a greater subjective symptom burden and lower scores indicating a lower burden. The asthma quality of life questionnaire (AQLQ) was used to evaluate changes in quality of life between T0 and T6 [29].

Response to biologic treatment between T0 and T6 was assessed using the FEOS scale [30], and asthma control was assessed using the multidimensional EXACTO scale, which classifies patients as: non-response, partial response, good response, and complete response/super-responder, based on exacerbations, ACT, systemic corticosteroids, and FEV1 changes relative to T0 [9].

IgE and peripheral blood eosinophils were measured as biomarkers at baseline and follow-up visits, as well as FeNO when available. Treatment adherence was strictly evaluated at each clinical visit in coordination with the Hospital Pharmacy Service.

### 2.7. Study Variables

The database included sociodemographic characteristics (age, sex, body mass index [BMI], smoking status, age at asthma onset, sensitization to perennial aeroallergens), IgE levels, specific IgE by RadioAllergoSorbent Test (RAST) and/or skin prick test, other biologics previously administered for SUA, and the presence of comorbidities (nasal polyposis, allergic rhinitis, gastroesophageal reflux disease [GERD], no previous allergy, rhinosinusitis [RS] with or without nasal polyposis [NP nonsteroidal anti-inflammatory drug-exacerbated respiratory disease [NSAID-NERD], obesity, obstructive sleep apnea [OSA], bronchiectasis). These were objectively assessed according to standardized definitions and finally confirmed with additional tests.

### 2.8. Statistical Analysis

For descriptive analysis, numerical variables were expressed as mean (X) ± standard deviation (SD), and frequency distributions as percentages. Qualitative variables were compared using the chi-square test or Fisher’s exact test. After applying the Kolmogorov–Smirnov test and assessing homogeneity and normality of variances, numerical variables were compared using Student’s *t*-test or the Mann–Whitney test (unpaired samples). Student’s *t*-test (paired samples) or the Wilcoxon test was used to assess response between T0 and T6 for the overall cohort and for the T2-high and T2-low groups. Between-group comparisons used Student’s *t*-test for independent samples when variables followed a normal distribution, and the Mann–Whitney test otherwise. A *p* value <0.05 was considered significant. Analyses were performed using SPSS version 18.0 (Chicago, IL, USA).

### 2.9. Ethical Considerations

The protocol was approved by the Drug Research Ethics Committee (CEIm) of University Hospital of Elda, with code 2023/04.

## 3. Results

A total of 33 patients were included (63.6% were analyzed retrospectively based on electronic medical records, while 36.4% were followed prospectively), with a median age of 56 (range, 50–68) years, median BMI of 27 (range, 24–31.5) kg/m^2^, 31 women and 2 men, total IgE of 50 (range, 15–117) IU/mL, median eosinophils of 350 (range, 185–490), median FeNO of 20 (range, 10–33) ppb, median ACT of 12 (range, 8–17), median ACQ of 4 (range, 3.4–7), with a median number of hospital admissions in the previous year of 0.5 (range, 0–2.5), median hospital length of stay of 2 (range, 1–12) days, median emergency visits of 4 (range, 2.3–5.8), median global exacerbations of 3 (range, 1–7), median OCS courses of 2 (range, 2–6), median FVC% of 84 (71–101), median FEV1% of 64 (range, 46–81.5) and median FEV1/FVC ratio of 63 (range, 55–76). The median time on tezepelumab was 9 (range, 6–12) months.

Of the 33 patients, 7 (22%) were allergic-eosinophilic, 13 (39%) eosinophilic, and 13 (39%) neutrophilic. For simplification, 20 patients (61%) were grouped as T2 phenotype and 13 (39%) as non-T2. Fifteen patients (45.5%) had received prior biologic therapy: eight (53%) from omalizumab, two (13%) from mepolizumab, four (27%) from benralizumab, and one (7%) from dupilumab.

**Figure 1:**
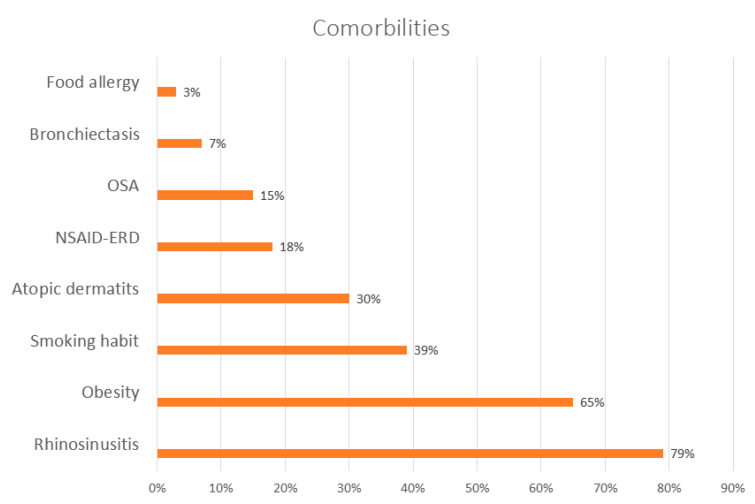
Overall comorbidity in the 28 patients who presented comorbidities. OSA: obstructive sleep apnea. NSAID-ERD: nonsteroidal anti-inflammatory drug-exacerbated respiratory disease.

Overall comorbidity was present in 28 patients (88%). Figure 1 shows the distribution of comorbidities, representing the proportion of patients with rhinosinusitis, obesity, smoking history, atopic dermatitis, polyposis, NERD, OSA, COPD, bronchiectasis, and food allergy. Of the total, 23 (70%) had negative RAST or prick tests, and 30% had sensitization to different aeroallergens, single or combined: mites, pollens, and epithelia. Comorbidities were distributed equally between the two groups, with no significant differences between patients with high and low T2, except for chronic rhinosinusitis with nasal polyposis. This condition was more prevalent in patients with the high T2 phenotype, especially the eosinophilic endotype.

Thirteen patients (39%) were former smokers; none were current smokers. Regarding medication, patients typically received one or more of the following: high-dose ICS (100%), LABA (100%), SABA (97%), LAMA (79%), leukotriene antagonists (76%), xanthines (12%), antihistamines (39%), and maintenance OCS (30%). In the previous year, 49% had been hospitalized, 97% had attended the emergency department, and 100% had received OCS courses, with 6.4 ± 4 severe exacerbations/year.

Table 1 shows differences in quantitative variables between the non-T2 and T2 groups at T0 (treatment initiation). The vast majority of compared quantitative variables did not show statistically significant differences. However, the baseline ACT score was lower in the T2 group (*p* < 0.05), reflecting poorer clinical asthma control. Regarding functional variables, absolute FVC was higher in the T2 group (*p* < 0.01), and absolute FEV1 and % predicted FEV1 were also higher (*p* < 0.05 for both), suggesting better lung function compared with the non-T2 group. In addition, absolute eosinophil count and FeNO were higher in the T2 group, as expected for its inflammatory phenotype (*p* < 0.001 and *p* < 0.01, respectively).

Table 2 shows post-treatment differences between both groups. Improvement in functional variables post-treatment was greater in the T2 phenotype compared with non-T2. This is reflected in the FEV1/FVC ratio, absolute FVC at one-year, absolute FEV1 (*p* < 0.01 for the three above), and % predicted FVC (*p* < 0.05). Regarding post-treatment FeNO, it was lower in the non-T2 group; however, despite decreasing in both groups, the reduction was greater in the T2 group (*p* < 0.01).

Table 3 shows pre- and post-treatment changes for the overall group, with a clear improvement in all clinical and functional variables. We highlight optimization in the number of hospital admissions, number of emergency visits, number of oral corticosteroid courses, ACT and ACQ scores, VAS for control, mini-AQLQ, absolute eosinophils, total exacerbations, and FeNO (*p* < 0.000). There was also a favorable evolution in maintenance OCS doses among those receiving them (*p* < 0.01), days of hospital stay (*p* < 0.001), FEV1/FVC ratio and absolute FEV1 (*p* < 0.05 for both), although there were no changes in IgE.

Table 4 compares pre- and post-treatment variables for the non-T2 group, highlighting an improvement in asthma control as expressed by ACT and ACQ scores, VAS, and mini-AQLQ (*p* < 0.000). In addition, the number of emergency visits and oral corticosteroid courses also decreased significantly (*p* < 0.000), together with a reduction in hospital admissions (*p* < 0.05). Finally, inflammatory markers such as absolute eosinophils (*p* < 0.000) and FeNO (*p* < 0.05) decreased, as did total exacerbations (*p* < 0.01).

Table 5 compares pre- and post-treatment values for the T2 group, showing significant improvement with reductions in hospital admissions and days of hospitalization (*p* < 0.01 for both), emergency visits (*p* < 0.000), and oral corticosteroid courses (*p* < 0.000). In addition, ACT and ACQ scores, VAS, mini-AQLQ, and biomarkers improved: eosinophils (*p* < 0.000) and FeNO (*p* < 0.001). A decrease in oral corticosteroid dose (*p* < 0.05), total exacerbations (*p* < 0.000), and an increase in absolute FEV1 (*p* < 0.05) were also observed.

To assess the potential confounding effect of prior biologic use, we performed a sub-analysis within the T2-high group comparing biologic-naïve vs. biologic-experienced patients. No statistically significant differences were found in exacerbations, oral corticosteroid dose, ACT, ACQ, mini AQLQ, VAS, biomarkers or lung function.

**Figure 2:**
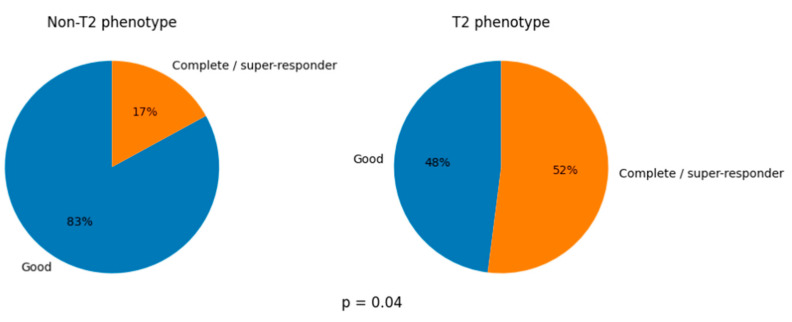
Percentage of patients with good response and complete response/super-responder in the T2 and non-T2 phenotypes.

When assessing post-treatment clinical response, there were nearly significant differences between non-T2 and T2 in FEOS (75 [74–81.3] vs. 76 [75–94.5], *p* = 0.07) (Table 2). When applying the EXACTO scale and comparing both groups, the non-T2 group showed a lower complete response (17%) compared with the T2 group (52%), although good response predominated in non-T2 (83%) compared with T2 (48%). This difference between groups was significant (*p* = 0.04) (Figure 2). Regarding the safety of the treatment, there have been no serious effects in patients. Only one patient experienced a transient adverse effect, which was pain at the puncture site. No patients discontinued treatment.

## 4. Discussion

Our study addresses the effect of tezepelumab in patients with SUA, including an extensive single-center series comparing its effectiveness in T2 and non-T2 patients. In fact, it is the only biologic treatment approved for non-T2 patients in whom it has been shown to be effective [13]. In our series, women predominated, as observed in other SUA series [31]. This has been attributed to factors such as greater exposure risk to domestic and occupational irritants, longer life expectancy, and a higher prevalence of anxiety and depression, which lead to greater healthcare use, more symptoms, and worse quality of life.

Notably, nearly half of patients underwent switching from other biologic therapies. Determinants of switching include both early and late therapeutic failure, phenotype changes, and adverse effects [32]. The most frequent prior biologic was omalizumab, which has been marketed for longer and is designed for a more allergic than eosinophilic profile. However, 30% had previously received anti-IL-5 or anti-IL-5 receptor therapies and even dupilumab, with a clear allergic profile.

The non-T2 phenotype has been scarcely studied and is often not analyzed in detail in large pivotal and real-world series [12,13,14,15,16,17]. In our study, we highlight a high number of non-T2 patients, most of whom were biologic-naïve. When comparing baseline characteristics between non-T2 and T2, the latter had better lung function but worse asthma control, with no differences in other severity parameters, and significant differences in inflammatory parameters (eosinophils and FeNO).

In the overall group analysis, pre- and post-treatment differences were found in all clinically relevant outcomes (severe exacerbations, hospital admissions, oral corticosteroids, asthma control, etc.) as well as lung function. Our findings are similar to clinical trials such as NAVIGATOR [14] and PATHWAY [15], which showed improvement regardless of phenotype or biomarkers.

In the group-specific analysis, the non-T2 phenotype showed less improvement in lung function, although all parameters related to asthma control, quality of life, and hospital reuse improved. This smaller lung function improvement may be due to patient complexity, severity, and likely airway remodeling, as well as possible delays in initiating biologic therapy. The T2 phenotype also improved across all parameters, with some lung function improvement, although with variable response, especially in FEV1 (mL), driven by very good response in some patients and minimal or no response in others. Likewise, many patients in this group were switchers and represent a poor-prognosis asthma subgroup per se, with an airway remodeling component as well.

Overall, all patients showed a good response measured by the FEOS scale, although improvement was greater in the T2 phenotype, with probably small clinical differences compared with the non-T2 group. When analyzing post-treatment clinical response using the EXACTO scale, there were differences in the percentage of complete responders/super-responders, with a lower percentage in non-T2, although 83% achieved good response in that group. Super-responders in the T2 phenotype reached 52%. This difference in complete response between the high T2 and low T2 phenotypes as well as the smaller improvement in lung function in patients with asthma with low T2 may be because asthma with high T2 responds significantly better to ICS and OCS than asthma with low T2. This is noteworthy given that many were switchers; therefore, tezepelumab contributed to stabilization and clinical remission. This supports the “do not be late” concept proposed in the literature [32].

Because TSLP is an epithelial cell-derived cytokine released in response to both T2 and non-T2 stimuli, its inhibition by tezepelumab explains its effectiveness in both phenotypes [14,15,16,17,18]. As previously mentioned, real-world studies on tezepelumab are scarce; our series is one of the largest reported in Spain with 33 patients. The remaining publications have small sample sizes [23,24,25], except for the recently published study by Khateeb et al. [22], which included 103 patients and found greater improvement in the high T2 phenotype, particularly among patients with eosinophil counts of at least 300 cells/μL. Another study by Miralles et al. included 53 patients from eight hospitals, and the results were similar to ours [26]. Our T2-low clinical remission results are also similar to the European multicenter series including 175 patients [21].

Our study has limitations inherent to its ambispective and observational design. As it was based in a single center, the results may have limited generalizability. Another limitation of our study is the evaluation of efficacy based on phenotype rather than endotype. Classifying patients by phenotype is a very superficial approach, and we should carry out an analysis based on endotypes (eosinophilic T2 or allergic T2) according to hypersensitivity mechanisms. We will take this into account in future work to assess the efficacy of tezepelumab according to endotype.

In conclusion, tezepelumab treatment proved effective in patients with uncontrolled severe asthma with both T2 and non-T2 phenotypes, significantly improving asthma control and quality of life in both groups. A substantial reduction was achieved in the need for oral corticosteroids and in maintenance and reliever medication, with a marked decrease in emergency visits and hospital admissions in both phenotypes. Exacerbations decreased dramatically across all patients. Clinical remission criteria were reached by 40% of the total cohort. Although lung function improvement measured by the FEOS scale was slightly lower in the non-T2 phenotype compared with T2, response was good in the vast majority of non-T2 patients, with 83% achieving good response. Regarding complete response or super-response, this was achieved by more than half of T2 patients, whereas the percentage was lower in non-T2 but with excellent outcomes in terms of good response. Lung function stabilized in both groups, with greater improvement in the T2 phenotype. These findings confirm that tezepelumab represents a valuable therapeutic option for the management of uncontrolled severe asthma regardless of inflammatory phenotype, with a favorable efficacy and safety profile that helps achieve therapeutic goals of disease control and reduction in systemic corticosteroid use and healthcare re-utilization.

## Figures and Tables

**Table 1:** Differences in quantitative variables between the non-T2 and T2 groups at T0 (treatment initiation). IQR: Interquartile Range. Ns: Non-significant. BMI: Body Mass Index. ACT: Asthma Control Test. ACQ: Asthma Control Questionnaire. AQLQ: Asthma Quality of Life Questionnaire. FEV1: Forced Expiratory Volume in 1 s. FVC: Forced Vital Capacity. IgE: Inmunoglobuline E. FeNO: Fractional Exhaled Nitric Oxide.

	Non-T2 Group	T2 Group	*p* Value
**Age, years (median [IQR])**	56 (50–69)	59 (49–66)	ns
**BMI, kg/m^2^ (median [IQR])**	28 (23.8–35.5)	27 (24–631)	ns
**Number of hospital admissions (median [IQR])**	0.5 (0–3)	0.5 (0–2)	ns
**Annual hospital admission rate (events/patient/year)**	0.7	0.8	ns
**Global exacerbations (median [IQR])**	6 (2–8)	7 (3–9)	ns
**Annual global exacerbation rate (events/patient/year)**	6.2	6.6	ns
**Days of hospital admission (median [IQR])**	3 (1–21)	4 (1–18)	ns
**Maintenance oral corticosteroid dose (median [IQR])**	11 (1.3–25)	8 (2–17)	ns
**Number of emergency visits (median [IQR])**	3 (2–8)	4 (3–5)	ns
**Annual emergency visit rate (events/patient/year)**	3.2	3.9	ns
**Number of oral corticosteroid cycles (median [IQR])**	4 (2–6)	3 (2–5)	ns
**Annual oral corticosteroid cycle rate (events/patient/year)**	4.1	3.3	ns
**ACT (median [IQR])**	14 (11.5–18)	10 (5–14)	<0.05
**ACQ (median [IQR])**	4.5 (3.4–6.4)	4 (3.5–7)	ns
**Mini AQLQ (median [IQR])**	2.3 (2–2.4)	2.4 (1.9–2.5)	ns
**VAS (median [IQR])**	7 (5.5–9)	7 (4–8)	ns
**FEV1/FVC ratio (median [IQR])**	70 (43–82)	67 (57–76)	ns
**FVC, mL(median [IQR])**	2075 (1522–2817)	2580 (2160–3265)	<0.01
**% FVC (median [IQR])**	75 (67–99)	90 (75.5–103)	=0.07
**FEV1, mL (median [IQR])**	1320 (850–2350)	1780 (1290–2415)	<0.05
**% FEV1 (median [IQR])**	66 (43–74.5)	67 (48–86)	<0.05
**Eosinophils, cells/μL (median [IQR])**	150 (120–207)	410 (330–605)	<0.001
**Total IgE, IU/mL (median [IQR])**	65 (25–86)	90 (24–215)	ns
**FeNO, ppb (median [IQR])**	12.5 (8.5–20)	28 (13–53)	<0.01

**Table 2:** Comparison of quantitative variables between the non-T2 and T2 groups post-treatment. IQR: Interquartile Range. Ns: Non-significant. BMI: Body Mass Index. ACT: Asthma Control Test. ACQ: Asthma Control Questionnaire. AQLQ: Asthma Quality of Life Questionnaire. FEV1: Forced Expiratory Volume in 1 s. FVC: Forced Vital Capacity. IgE: Inmunoglobuline E. FeNO: Fractional Exhaled Nitric Oxide.

	Non-T2 Group	T2 Group	*p* Value
**Number of hospital admissions (median [IQR])**	0 (0–0)	0 (0–0)	ns
**Annual hospital admission rate (events/patient/year)**	0.2	0.3	ns
**Global exacerbations (median [IQR])**	0 (0–1)	0 (0–1)	ns
**Annual global exacerbation rate (events/patient/year)**	0.7	0.8	ns
**Days of hospital admission (median [IQR])**	0 (0–0)	0 (0–0)	ns
**Maintenance oral corticosteroid dose (median [IQR])**	2 (0–3.5)	0 (0–1.3)	ns
**Number of emergency visits (median [IQR])**	0 (0–1)	0 (0–0)	ns
**Annual emergency visit rate (events/patient/year)**	0.4	0.6	ns
**Number of oral corticosteroid cycles (median [IQR])**	0 (0–1)	0 (0–1)	ns
**Annual oral corticosteroid cycle rate (events/patient/year)**	0.6	0.8	ns
**ACT (median [IQR])**	23.5 (20–24.75)	22 (21.5–23)	ns
**ACQ (median [IQR])**	0.7 (0.1–1.3)	0.8 (0.4–1.2)	ns
**Mini AQLQ (median [IQR])**	5 (4.4–5.9)	5.3 (4.6–5.7)	ns
**VAS (mean ± SD)**	2 (2–3)	2 (1.5–3)	ns
**FEV1/FVC ratio (median [IQR])**	64.5 (45.5–83)	74 (56.5–80)	<0.01
**FVC, mL(median [IQR])**	2125 (1378–2882)	2530 (2180–3265)	<0.01
**% FVC (median [IQR])**	73 (49–102)	89 (82–102)	<0.05
**FEV1, mL (median [IQR])**	1090 (878–2450)	1915 (1305–2550)	<0.01
**% FEV1 (median [IQR])**	54 (37–85.5)	79 (54–90.5)	ns
**Absolute FEV1 difference, mL (median [IQR])**	180 (140–225)	175 (128–260)	ns
**Eosinophils, cells/μL (median [IQR])**	100 (65–137.5)	180 (55–250)	ns
**Total IgE, IU/mL (median [IQR])**	42 (22–71)	49 (28–76)	ns
**FeNO, ppb (median [IQR])**	9 (8–17.5)	18 (9–29.5)	<0.01
**FEOS (median [IQR])**	75 (74–81.3)	76 (75–94.5)	=0.07

**Table 3:** Changes between pre-treatment and post-treatment for the overall group. IQR: Interquartile Range. Ns: Non-significant. BMI: Body Mass Index. ACT: Asthma Control Test. ACQ: Asthma Control Questionnaire. AQLQ: Asthma Quality of Life Questionnaire. FEV1: Forced Expiratory Volume in 1 s. FVC: Forced Vital Capacity. IgE: Inmunoglobuline E. FeNO: Fractional Exhaled Nitric oxide.

	Pre-Treatment	Post-Treatment	*p* Value
**Number of hospital admissions (median [IQR])**	0.5 (0–2.5)	0 (0–0)	<0.000
**Annual hospital admission rate (events/patient/year)**	0.8	0.2	<0.000
**Global exacerbations (median [IQR])**	6 (2–9)	0 (0–1)	<0.000
**Annual global exacerbation rate (events/patient/year)**	6.5	0.7	<0.000
**Days of hospital admission (median [IQR])**	3 (1–12)	0 (0–0)	<0.001
**Maintenance oral corticosteroid dose (median [IQR])**	9.5 (2.5–15)	0.5 (0–2)	<0.01
**Number of emergency visits (median [IQR])**	3.5 (2.3–5.8)	0 (0–1)	<0.000
**Annual emergency visit rate (events/patient/year)**	3.7	0.2	<0.000
**Number of oral corticosteroid cycles (median [IQR])**	4 (2–6)	0 (0–1)	<0.000
**Annual oral corticosteroid cycle rate (events/patient/year)**	3.7	0.7	<0.000
**ACT (median [IQR])**	12 (8–17)	23 (21.5–24)	<0.000
**ACQ (median [IQR])**	4 (3.4–7)	0.6 (0.3–1.2)	<0.000
**Mini AQLQ (median [IQR])**	2.4 (2–2.5)	5.1 (4.4–5.7)	<0.000
**VAS (median [IQR])**	7 (4–8)	2 (2–3)	<0.000
**FEV1/FVC ratio (median [IQR])**	63 (55–76)	71 (54.5–80)	<0.05
**FVC, mL (median [IQR])**	2540 (1885–3125)	2490 (1980–3150)	ns
**% FVC (median [IQR])**	84 (71–101)	86 (64.5–102)	ns
**FEV1, mL (median [IQR])**	1480 (1060–2415)	1730 (1095–2520)	<0.05
**% FEV1 (median [IQR])**	64 (46–81.5)	73 (49.5–89)	ns
**Eosinophils, cells/μL (median [IQR])**	350 (185–490)	110 (60–230)	<0.000
**Total IgE, IU/mL (median [IQR])**	50 (15–117)	45 (22–74)	ns
**FeNO, ppb (median [IQR])**	20 (10–33)	14 (8–23)	<0.000

**Table 4:** Comparison between pre-treatment and post-treatment for the non-T2 group. IQR: Interquartile Range. Ns: Non-significant. BMI: Body Mass Index. ACT: Asthma Control Test. ACQ: Asthma Control Questionnaire. AQLQ: Asthma Quality of Life Questionnaire. FEV1: Forced Expiratory Volume in 1 s. FVC: Forced Vital Capacity. IgE: Inmunoglobuline E. FeNO: Fractional Exhaled Nitric Oxide.

	**Pre-Treatment**	**Post-Treatment**	***p*** **Value**
**Number of hospital admissions (median [IQR])**	0.5 (0–3)	0 (0–0)	<0.05
**Annual hospital admission rate (events/patient/year)**	0.7	0.2	<0.05
**Global exacerbations (median [IQR])**	6 (2–8)	0 (0–1)	<0.01
**Annual global exacerbation rate (events/patient/year)**	6.2	0.7	<0.000
**Days of hospital admission (median [IQR])**	3 (1–21)	0 (0–0)	=0.07
**Maintenance oral corticosteroid dose (median [IQR])**	11 (1.3–25)	2 (0–3.5)	ns
**Number of emergency visits (median [IQR])**	3 (2–8)	0 (0–1)	<0.000
**Annual emergency visit rate (events/patient/year)**	3.2	0.4	<0.000
**Number of oral corticosteroid cycles (median [IQR])**	4 (2–6)	0 (0–1)	<0.000
**Annual oral corticosteroid cycle rate (events/patient/year)**	4.1	0.6	<0.000
**ACT (median [IQR])**	14 (11.5–18)	23.5 (20–24.75)	<0.000
**ACQ (median [IQR])**	4.5 (3.4–6.4)	0.7 (0.1–1.3)	<0.000
**Mini AQLQ (median [IQR])**	2.3 (2–2.4)	5 (4.4–5.9)	<0.000
**VAS (median [IQR])**	7 (5.5–9)	2 (2–3)	<0.000
**FEV1/FVC ratio (median [IQR])**	70 (43–82)	64.5 (45.5–83)	=0.06
**FVC, mL (median [IQR])**	2075 (1522–2817)	2125 (1378–2882)	ns
**% FVC (median [IQR])**	75 (67–99)	73 (49–102)	ns
**FEV1, mL (median [IQR])**	1320 (850–2350)	1090 (878–2450)	ns
**% FEV1 (median [IQR])**	66 (43–74.5)	54 (37–85.5)	ns
**Eosinophils, cells/μL (median [IQR])**	150 (120–207)	100 (65–137.5)	<0.000
**Total IgE, IU/mL (median [IQR])**	65 (25–86)	42 (22–71)	ns
**FeNO, ppb (median [IQR])**	12.5 (8.5–20)	9 (8–17.5)	<0.05

**Table 5:** Comparison between pre-treatment and post-treatment for the T2 group. IQR: Interquartile range. Ns: Non-significant. BMI: Body Mass Index. ACT: Asthma Control Test. ACQ: Asthma Control Questionnaire. AQLQ: Asthma Quality of Life Questionnaire. FEV1: Forced Expiratory Volume in 1 s. FVC: Forced Vital Capacity. IgE: Inmunoglobuline E. FeNO: Fractional Exhaled Nitric Oxide.

	Pre-Treatment	Post-Treatment	*p* Value
**Number of hospital admissions (median [IQR])**	0.5 (0–2)	0 (0–0)	<0.01
**Annual hospital admission rate (events/patient/year)**	0.8	0.3	<0.05
**Global exacerbations (median [IQR])**	7 (3–9)	0 (0–1)	<0.000
**Annual global exacerbation rate (events/patient/year)**	6.6	0.8	<0.05
**Days of hospital admission (median [IQR])**	4 (1–18)	0 (0–0)	<0.01
**Maintenance oral corticosteroid dose (median [IQR])**	8 (2–17)	0 (0–1.3)	<0.05
**Number of emergency visits (median [IQR])**	4 (3–5)	0 (0–0)	<0.000
**Annual emergency visit rate (events/patient/year)**	3.9	0.6	<0.05
**Number of oral corticosteroid cycles (median [IQR])**	3 (2–5)	0 (0–1)	<0.000
**Annual oral corticosteroid cycle rate (events/patient/year)**	3.3	0.8	<0.05
**ACT (median [IQR])**	10 (5–14)	22 (21.5–23)	<0.000
**ACQ (median [IQR])**	4 (3.5–7)	0.8 (0.4–1.2)	<0.000
**Mini AQLQ (median [IQR])**	2.4 (1.9–2.5)	5.3 (4.6–5.7)	<0.000
**VAS (median [IQR])**	7 (4–8)	2 (1.5–3)	<0.000
**FEV1/FVC ratio (median [IQR])**	67 (57–76)	74 (56.5–80)	ns
**FVC, mL (median [IQR])**	2580 (2160–3265)	2530 (2180–3265)	ns
**% FVC (median [IQR])**	90 (75.5–103)	89 (82–102)	ns
**FEV1, mL (median [IQR])**	1780 (1290–2415)	1915 (1305–2550)	<0.05
**% FEV1 (median [IQR])**	67 (48–86)	79 (54–90.5)	ns
**Eosinophils, cells/μL (median [IQR])**	410 (330–605)	180 (55–250)	<0.000
**Total IgE, IU/mL (median [IQR])**	90 (24–215)	49 (28–76)	ns
**FeNO, ppb (median [IQR])**	28 (13–53)	18 (9–29.5)	<0.001

## Data Availability

The original contributions presented in this study are included in the article. Further inquiries can be directed to the corresponding author.

## Abbreviations

The following abbreviations are used in this manuscript:

SUA	Severe uncontrolled asthma
OCS	Oral glucocorticoids
ICS	Inhaled glucocorticoids
LABAs	Long-acting β-adrenergic agonists
TSLP	Thymic stromal lymphopoietin
FeNO	Fractional exhaled nitric oxide
GEMA	Spanish guideline on the management of asthma
LAMA	Long-acting muscarinic antagonist
SABA	Short-acting β2-agonist
FEV1	Forced expiratory volume in 1 s
SEPAR	Spanish Society of Pulmonology and Thoracic Surgery
ACT	Asthma control test
ACQ	Asthma control questionnaire
VAS	Visual analog scale
AQLQ	Asthma quality of life questionnaire
RAST	RadioAllergoSorbent test
RS	Rhinosinusitis
NP	Nasal polyposis
NSAID-NERD	Nonsteroidal anti-inflammatory drug-exacerbated respiratory disease
OSA	Obstructive sleep apnea
CEIm	Drug Research Ethics Committee
